# Supplementary material for: Effective Remediation of Arsenic-Contaminated Soils by EK-PRB of Fe/Mn/C-LDH: Performance, Characteristics, and Mechanism
Source: Int J Environ Res Public Health. 2022 Apr 6;19(7):4389. doi: 10.3390/ijerph19074389 (PMC8998996; doi:10.3390/ijerph19074389)
Supplement: Supplementary file 1 [file ijerph-19-04389-s001.zip › ijerph-1606092-supplementary.pdf]

Effective Remediation of Arsenic-Contaminated Soils by EK-PRB of Fe/Mn/C-LDH: Performance, Characteristics, and Mechanism

Zongqiang Zhu [1,2,3], Shuai Zhou [1,2], Xiaobin Zhou [1,2,*], Shengpeng Mo [1,2], Yinian Zhu [1,2], Lihao Zhang [1,2], Shen Tang [1,2], Zhanqiang Fang [4], Yinming Fan[1,2,*]

1 Collaborative Innovation Center for Water Pollution Control and Water Safety in Karst Area, Guilin University of Technology, Guilin 541004, China

2 The Guangxi Key Laboratory of Theory and Technology for Environmental Pollution Control, Guilin University of Technology, Guilin 541004, China

3 Technical Innovation Center of Mine Geological Environmental Restoration Engineering in Southern Karst Area, Nanning 530022, China

4 School of Chemistry and Environment, South China Normal University, Guangzhou 510006, China

* Correspondence: author: Yming_Fan2015@163.com; zhouxiaobin@glut.edu.cn

1. Catalysts characterization

The crystal structure of the samples was acquired by X-ray powder diffraction (XRD) on a D8 instrument (BrukerAXS) with Cu Kα radiation (40 kV, 40 mA, scanning step = 0.02). The surface morphologies and microstructures of the catalysts were respectively characterized via the scanning electron microscopy (SEM, Hitachi) with an energy dispersive X-ray attachment (EDS). Fourier transform infrared absorption spectrometer (Thermo Nexus 470FT-IR) was used to determine the functional groups contained in the synthesized Sr-HAP and verify the structure of the sample. Zeta potential analyzer (Zetasizer Nano ZS90) was used to measure the zero point potential.

2. Supplementary tables

Table S1. The physical and chemical properties of soil before and after pollution

Soil	pH	Organic matter (g/kg)	Alkali hydrolyzed nitrogen (mg/kg)	Available phosphorus (mg/kg)	Target arsenic concentration (mg/kg)
Background soil	5.35	25.31	91.35	9.01	—
Arsenic contaminated soil	6.35	—	—	—	500

* The selection of target heavy metal pollution concentration was based on the field investigation data of Guangxi contaminated site remediation project;
* "—" represented not detected.

Table S2. The extraction of heavy metals from soil by improved BCR method

Arsenic form	Extraction	experimental condition	soil/water
Acid dissolved state	20mL 0.1 M HOAc	Oscillated at 25 °C for 16h	1:40
Reducible state	20mL 0.5 M NH_4OH•HCl	Oscillated at 25 °C for 16h	1:40
Oxidizable state	10mL 8.8 M H_2O_2<br>50mL1 M NH_4OAc	Water bath heating at 85°C	1:10
Residue state	HCl~HNO_3~HF~$HClO_4$	Electric heating plate digestion	——

Table S3. The weight percentage of elements in Fe/Mn/C-LDH

Material	Elements	Spectrum
Fe/Mn/C-LDH	C	26.59
	O	35.56
	Mn	24.52
	Fe	13.33

3. Supplementary figures

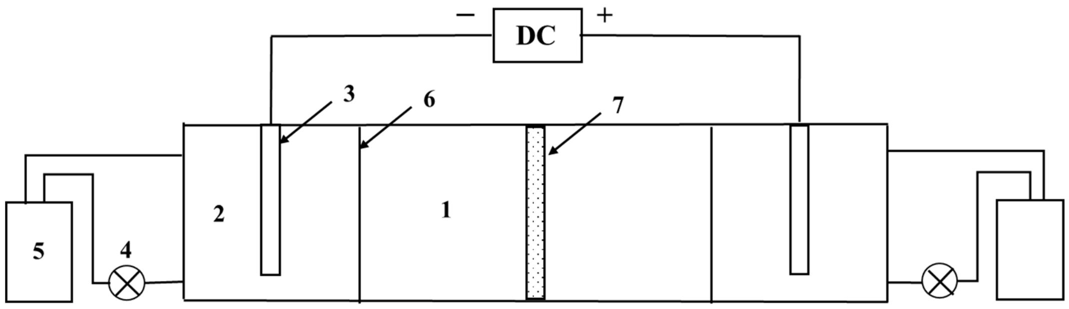

(1) Soil chamber (2) Electrolytic chamber (3) Electrode (4) Circulating pump

(5) Collecting tank (6) Division plate (7) PRB filler Fe/Mn/C-LDH

Figure S1. Device diagram of electrically driven enhanced permeable reaction wall.
